# An Important Finding of White Matter Injury in Late Preterm Infant: Deep Medullary Vein Involvement

**DOI:** 10.3389/fped.2020.597567

**Published:** 2020-12-17

**Authors:** Dan Chen, Jing Sun, Qiuyu Li, Wenjuan Bai, Jian Mao

**Affiliations:** Department of Pediatrics, Shengjing Hospital of China Medical University, Shenyang, China

**Keywords:** deep medullary vein, white matter injury, late preterm infant, PVL, high risk factor

## Abstract

**Objective:** To investigate high risk factors and magnetic resonance imaging (MRI) features in late preterm infants with severe white matter injury (WMI) associated with abnormal deep medullary veins (DMVs).

**Materials and Methods:** Preterm infants with severe WMI, who were hospitalized in Shengjing Hospital from 1st January 2009 to 31st December 2018, were enrolled in this retrospective study. High risk factors and MRI characteristics of infants with abnormal DMVs were analyzed and compared with those of infants without DMV abnormalities.

**Results:** A total of 2032 late preterm infants were examined by MRI; 71 cases (3.5%) had severe WMI and 15 of these (21.1%) had abnormal DMVs. The incidence of maternal diabetes was higher in infants with abnormal DMVs and neonatal convulsions were more likely (*P* < 0.05). The incidence of grade IV injury (*P* < 0.05), white matter periventricular cysts and thalamic injury (*P* < 0.01), cerebral venous sinus thrombus (*P* < 0.01) and germinal matrix/intraventricular hemorrhage (*P* < 0.05) were higher in infants with abnormal DMVs than in infants with normal DMVs.

**Conclusions:** Congestion/thrombosis of DMVs may be an important cause of severe WMI in late preterm infants, especially in periventricular leukomalacia-like WMI. WMI with abnormal DMVs is more likely to lead to thalamic injury.

## Introduction

At 34 weeks, the brain weight of preterm infants is only 65% that of term infants, and the cortex volume is 53% that of term infants (1). Damage at this stage of development will also change the trajectory of specific processes in the development of neurons and glial cells, resulting in neurological dysfunction in survivors (1). The incidence of cerebral palsy in late preterm infants is three times higher than in term infants, (2) and about 25% lag behind term infants in learning, language and other neurodevelopment (3). At 34–37 weeks of gestation, oligodendrocytes are still late oligodendrocyte precursors and vascular development of the white matter area is immature, making the brain more prone to white matter injury (WMI) (1, 4–7).

WMI, which is damage of neurovascular units, involves oligodendrocytes, axons and vascular endothelial cells. Research has focused mainly on oligodendrocytes and axons, with fewer studies looking at vascular factors. The deep medullary veins (DMVs) are the veins of white matter, which drain to the corresponding subependymal vein and finally connect to the deep venous system. There are four confluence areas in the subependymal vein of DMVs, which is unique among medullary veins. Over the past decade, the characteristic patterns of DMV thrombosis and peripheral tissue infarction have been increasingly recognized using neuroimaging (8–10). The use of early magnetic resonance imaging (MRI) enables us to observe the special pattern of white matter lesions in which DMVs are involved. When the lesions are distributed in parallel or radially in the deep white matter area, diseases associated with DMVs should be suspected. In premature infants, the immature deep venous system is more prone to venous congestion and thrombosis (11).

There are few studies on DMV lesions in premature infants with WMI and there is no comparative study with other white matter lesions. Late preterm infants are the main group of preterm infants that can be examined by MRI in the early stages. In this study, we investigated high risk factors and MRI characteristics of late preterm infants with DMV-associated WMI.

## Materials and Methods

### Patients

Late preterm infants who were hospitalized in Shengjing Hospital from 1st January 2009 to 31st December 2018 and met the following inclusion criteria: (1) MRI head examination with informed consent of guardian; (2) age 34–36^+6^ weeks; (3) severe WMI. Other encephalopathies or congenital abnormality of brain development were excluded.

The infants were divided into two groups: those with abnormal DMVs and those with normal DMVs.

Risk factors prompting MRI evaluation included: (1) premature rupture of fetal membrane, intrauterine distress or placental abruption before delivery; (2) asphyxia, resuscitation and rescue history, circulatory dysfunction and infection during or after delivery; and (3) early convulsions.

This study was approved by the ethics committee of Shengjing Hospital.

### Assessment of Brain Injury

MRI scans were analyzed by a neuroradiologist, a pediatric neurologist and a neonatologist who were unfamiliar with the clinical history. WMI classification was carried out as described by Martinez-Biarge et al. (12), with some improvements. Cases in grade II with clearly abnormal signals in the corpus callosum were included in grade III and hemorrhagic injury was not excluded. Severe WMI was classified as Grade III or grade IV.

DMV abnormalities were characterized by congestion/thrombosis of DMVs, usually appear as linear, radial, fan-shaped low signal lesions, close to the anatomic location of the DMV in the T2 weighted image (8).

### Collection of Clinical Data

Data, including delivery by cesarean section, gestational hypertension, diabetes mellitus, premature rupture of membranes and placental abruption, were collected for the mothers. Gestational age, weight, gender, whether small for gestational age, Apgar score, resuscitation history, circulatory disorders, early-onset sepsis, convulsions, and MRI data were collected for the newborns.

History of resuscitation and rescue refers to positive pressure ventilation, tracheal intubation, chest compression or epinephrine application during postnatal resuscitation; circulatory disorders include at least two of the following indicators: prolonged capillary filling time (Heel > 5 s), hypotension (<10th centile for birth weight and postnatal age), oliguria (<1 ml/kg per hour), increased heart rate (>160/min) and increased liver (>2 cm below the costal margin of the midline of the right clavicle). Early-onset sepsis, defined by the National Institute of Child Health and Human Development and Vermont Oxford Networks as sepsis with onset at ≤3 days of age (13). Convulsion refers to the movement convulsion of the whole body or a part of the body muscle, which is caused by the involuntary strong contraction of the skeletal muscle.

### Instrumentation

MRI of the head was performed using an Intera Achieva 3.0T MRI system (Philips, Best, Netherlands). All infants were scanned by conventional MRI and diffusion-weighted imaging (DWI). Because of the retrospective study design, there are differences in imaging schemes, sequences and parameters measured.

### Statistical Analysis

SPSS 19.0 statistical software was used for statistical analysis, which involved the application of the *t*-test, the Mann Whitney *U* test, the χ^2^ test, and Fisher's exact test. Multivariate logistic regression analysis was used to analyze clinical data. *P* < 0.05 was taken as statistically significant.

## Results

### General Data

MRI was performed on 2,032 late preterm infants, 71 (3.5%) of whom had severe WMI, all the severe patients survived during hospitalization. Of these, 15 cases (21.1%) had abnormal DMVs and 56 cases had normal DMVs.

### Clinical Characteristics of DMVs-Related WMI

The incidence of maternal diabetes was higher in infants with abnormal DMVs (*P* < 0.05) and the Apgar score 1 min after birth was lower (*P* < 0.05). Multivariate logistic regression analysis confirmed a significant difference in the incidence of maternal diabetes between the two groups (*P* = 0.018; 95% CI 1.363–25.858), but showed no significant difference in Apgar score at 1 min (*P* = 0.124; 95% CI 0.944–1.622). The incidence of convulsions was higher in infants with abnormal DMVs (*P* < 0.05, Table 1).

**Table 1:** Comparison of case characteristics between the two groups.

	**Abnormal DMVs ** ***n* = 15**	**Normal DMVs** ***n* = 56**	***t/z/χ^2^***	***P***
**Pregnant mother**				
Cesarean section	11 (15)	36 (56)	0.433	0.511
Gestational hypertension	4 (15)	8 (56)	–	0.264^#^
Gestational diabetes	5 (15)	5 (56)	–	0.029^#^
Premature rupture of membranes	7 (15)	28 (56)	0.053	0.819
Placental abruption	2 (15)	1 (56)	–	0.111^#^
**Newborn**				
Gestational age (weeks)	35.3 ± 0.9	35.2 ± 0.9	0.487	0.628
Weight	2400.0 ± 589.1	2297.4 ± 356.8	0.644	0.528
Gender (male/female)	11/4	33/23	1.042	0.307
Small for gestational age	4 (15)	8 (56)	–	0.264^#^
Apgar score (1 min)	7 (6,8)	8 (7,10)	−1.965	0.049^*^
Apgar score (5 min)	9 (9,9)	9 (8,10)	−0.944	0.345^*^
Resuscitation and rescue	7 (15)	19 (56)	0.827	0.363
Circulatory disorders	1 (15)	5 (56)	–	1.000^#^
Early sepsis	6 (15)	8 (56)	–	0.061^#^
Convulsions	5 (15)	5 (56)	–	0.029^#^

* *Mann Whitney U test*.

# *Fisher's exact test*.

### MRI Features of DMV-Related WMI

#### Manifestations of Abnormal DMVs

**Figure 1:**
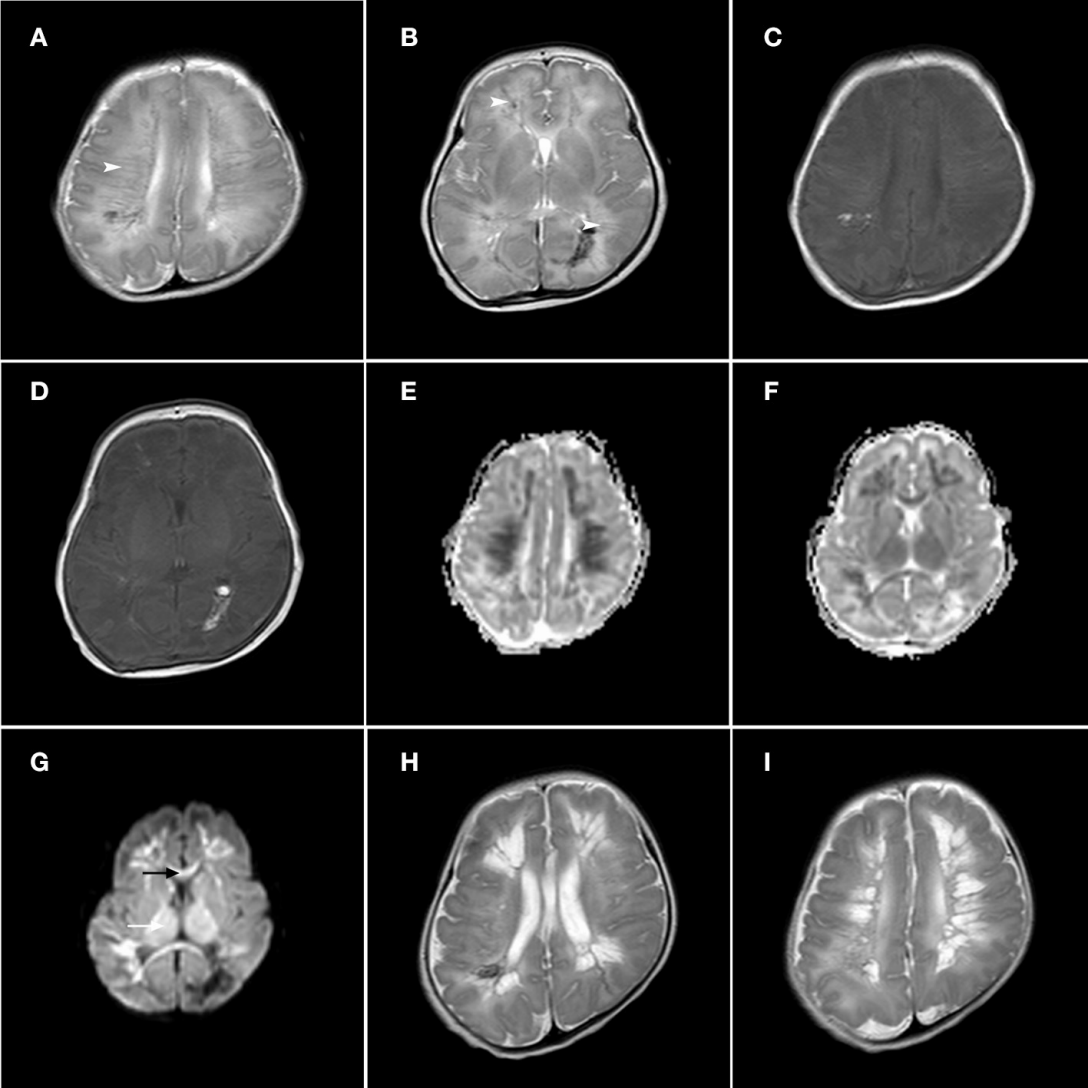
Axial T1-weighted image, axial T2-weighted image, axial ADC maps and DWI sections in a late premature neonate (gestational age, 35+5 weeks; **(A–G)**: 8 days after birth, **(H,I)**: 21 days after birth). The T2-weighted images show linear and radial lesions in the frontal parietal area **(A)** and prefrontal area and peritrigonal area **(B)**, with low signal changes (arrow head). The T1-weighted signal did not change significantly **(C,D)**. Axial ADC maps showed a significant decrease in ADC value in the white matter area around the linear injury **(E,F)**. In DWI **(G)**, the signals of the corpus callosum (black arrow) and thalamus (white arrow) were increased. On the 21st day after birth, the second T2-weighted MRI scan showed the formation of cystic changes in the prefrontal region, trigonal region **(H)** and frontal parietal region **(I)**.

**Figure 2:**
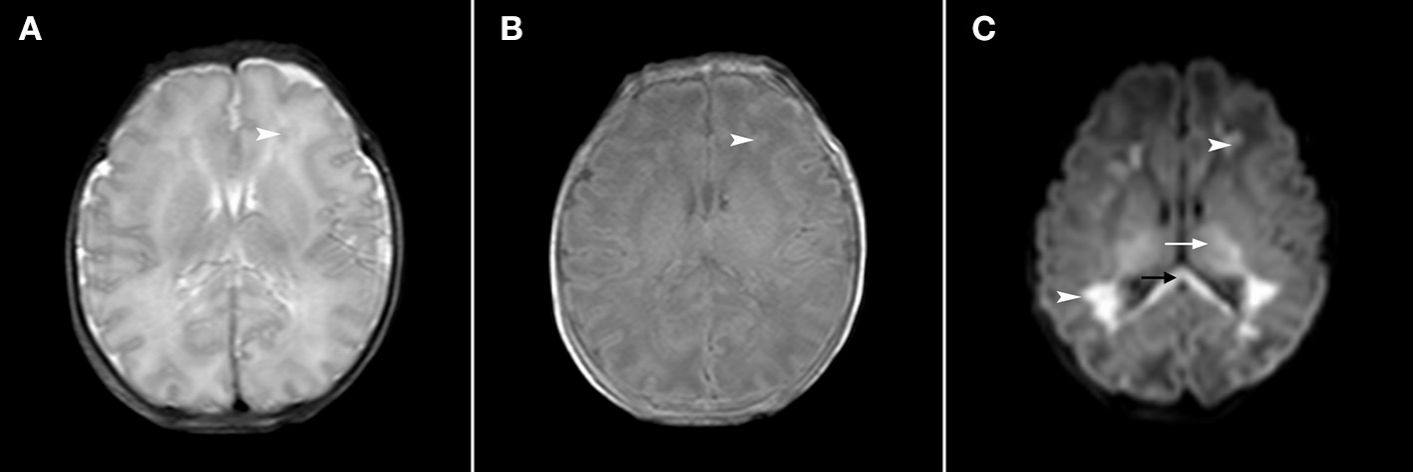
Axial T2-weighted, axial T1-weighted and DWI sections in a late premature neonate (gestational age, 35+5 weeks; 2 days after birth). In the T2-weighted image **(A)**, the white matter signal of the prefrontal area was slightly decreased (arrowhead) and in the T1-weighted image **(B)**, the signal was slightly increased (arrowhead). In the DWI **(C)**, the signals of the prefrontal area and trigonometry were significantly increased (arrowhead), and the signals of the corpus callosum (black arrow) and thalamus (white arrow) were also significantly increased.

The first MRI scan of infants with abnormal DMVs was 1–18 days after birth, with an average age of 6.7 ± 4.9 days. DMV abnormalities were more significant in T2 weighted images, with obvious linear, radial, and fan-shaped low signal lesions, whereas there were no obvious changes in the T1 images. On the apparent diffusion coefficient (ADC) map, there was a significant decrease in the ADC value of white matter around the linear abnormality. Lesions of the corpus callosum and the posterior limb of the internal capsule and gray matter, such as thalamic injury, which produce changes in high intensity signals, were more obvious in early DWI, with no obvious changes in the T1- and T2-weighted images (Figure 1). Compared with WMI associated with DMV abnormalities, WMI without DMV abnormalities showed no obvious characteristic signal changes in the T2-weighted images and the lesions were more apparent in DWI (Figure 2).

#### Comparison of MRI Features Between Infants With Abnormal and Normal DMVs

There was no significant difference between the two groups in the time or gestational age of the first MRI examination. The incidence of grade IV injury was higher in infants with abnormal DMVs (*P* < 0.05) and periventricular leukomalacia (PVL)-like WMI was more common (*P* < 0.01). Injury in all cases in both groups involved the frontal and parietal white matter; the incidence of peritrigonal involvement was higher in infants with normal DMVs (*P* < 0.01). There was no significant difference between the two groups in the incidence of involvement of corpus callosum or posterior limb of the internal capsule. With respect to gray matter injury, infants with abnormal DMVs were more prone to thalamic injury (*P* < 0.01). The incidence of both cerebral sinovenous thrombosis (CSVT) (*P* < 0.01) and germinal matrix-intraventricular hemorrhage (GM-IVH) (*P* < 0.05) were higher in the group with abnormal DMVs (Table 2).

**Table 2:** Comparison of initial MRI features between the two groups.

	**Abnormal DMVs** ***n* = 15**	**Normal DMVs** ***n* = 56**	***t/χ^2^***	***P***
Inspection time (days from birth)	6.7 ± 4.9	7.9 ± 3.4	−1.071	0.288
Gestational age at examination (weeks)	36.5 ± 1.2	36.3 ± 1.0	0.462	0.646
Grade IV injury	6 (1)	6 (56)	–	0.015^#^
PVL-like WMI	6 (15)	4 (56)	–	0.004^#^
**WMI distribution**				
Prefrontal	15 (15)	50 (56)	–	0.331^#^
Frontal parietal	15 (15)	56 (56)		
Peritrigonal	11 (15)	55 (56)	–	0.006^#^
Temporal	2 (15)	6 (56)	–	0.673^#^
Corpus callosum	13 (15)	47 (56)	–	1.000^#^
Posterior limb of internal capsule	9 (15)	26 (56)	0.872	0.350
**Distribution of gray matter injury**				
Thalamus	8 (15)	4 (56)	–	<0.001^#^
Pontine	2 (15)	4 (56)	–	0.6^#^
**Hemorrhagic injury**				
GM-IVH	5 (15)	6 (56)	–	0.047^#^
CSVT	3 (15)	0 (56)	–	0.008^#^

# *Fisher's exact test*.

*PVL, periventricular leukomalacia; WMI, white matter injury; GM-IVH, germinal matrix-intraventricular hemorrhage; CSVT, sinovenous thrombosis*.

#### PVL-Like WMI Associated With DMVs

**Figure 3:**
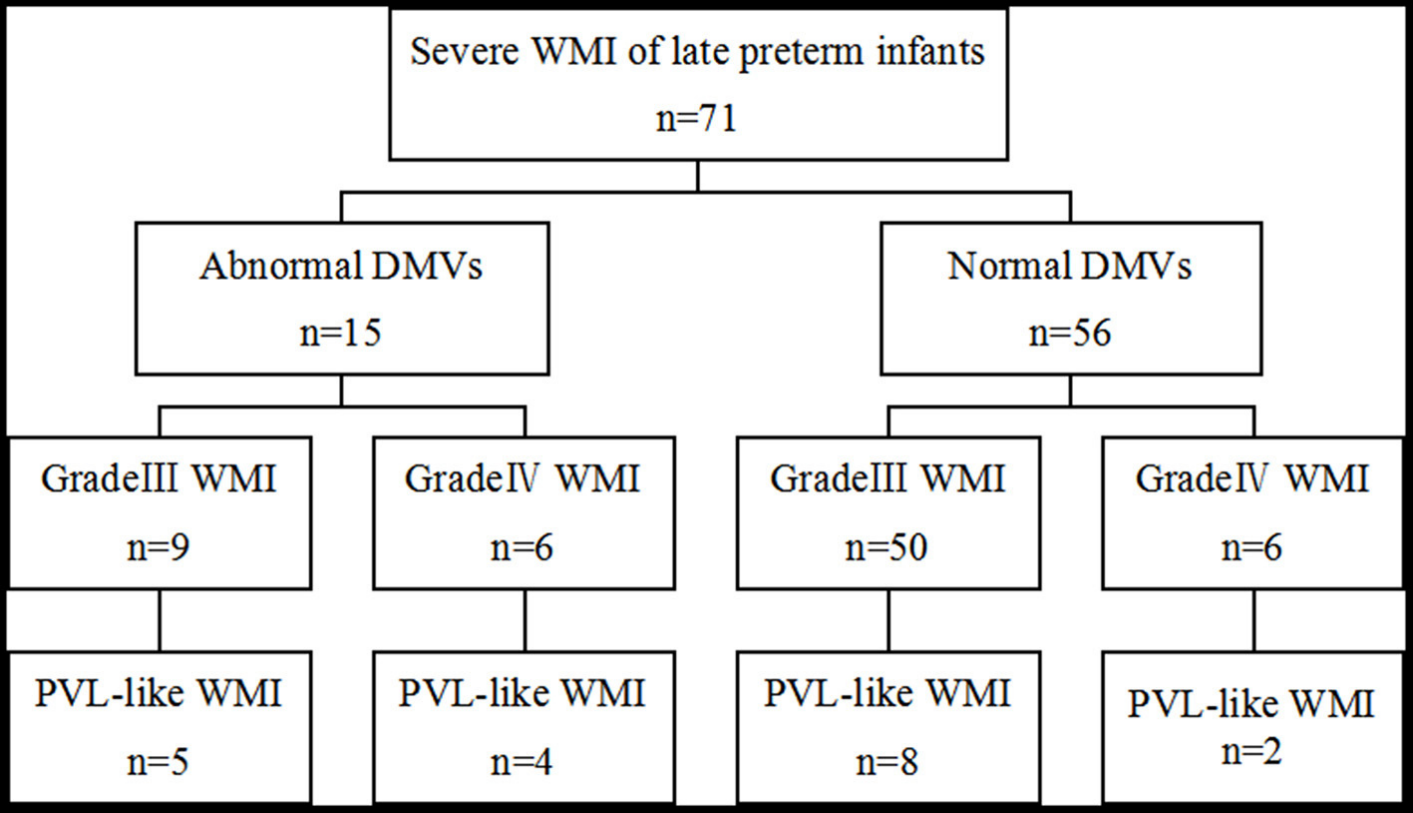
Characteristics of PVL-like WMI in two groups.

PVL-like WMI was found at the first MRI scan in six infants with abnormal DMVs. The examination took place seven days after birth in two cases, 8–14 days in two cases, and 15–21 days in two cases. A second head MRI examination was performed 15–21 days after birth in five cases. All of these showed PVL-like WMI, although three had no PVL-like WMI at the first examination. PVL was found by MRI in nine cases (60%). PVL-like WMI was seen in the first MRI scan in four cases with normal DMVs; the examination took place 8–14 days after birth in three 3 cases and 15–21 days in one case. A second MRI was performed in 25 cases. Eight cases had PVL-like WMI, which was not observed in the first scan in six cases. Four cases were examined after 15–21 days and two cases were examined after 22–28 days. Two MRI scans in 10 cases (17.9%) showed PVL-like WMI (Figure 3).

## Discussion

This is the first study to investigate the involvement of DMVs in WMI in late preterm infants. High risk factors and MRI characteristics of WMI associated with abnormal DMVs were analyzed and compared with other WMI. Congestion/thrombosis of DMVs was an important factor in 21.1% of cases with severe WMI, which mainly involved white matter in the prefrontal and frontal parietal areas and rarely that in the temporal area. WMI associated with DMV abnormalities was more severe than other WMI; PVL-like changes were more likely and thalamic injury was more common.

Previous imaging studies suggested that congestion/ thrombosis of blood vessels in the DMV distribution area may form part of the pathological chain leading to WMI around the ventricles in neonates (8). When the DMVs are congested, serious cerebral venous thrombosis and secondary white matter damage around the ventricles may occur (14). Post-mortem angiography showed that the fan-shaped drainage area in the confluent veins was very consistent with the PVL area, with edema and hemorrhage due to filling of the DMVs (15). The pathogenesis of DMV abnormalities in neonates is unclear. In preterm infants, germinal matrix bleeding may lead to terminal vein occlusion or medullary vein congestion (8, 16), and DMV thrombosis may be associated with perivenous hemorrhage in preterm infants (17, 18). Subependymal hemorrhage and subsequent local thrombus/hematoma may increase upstream venous drainage pressure, creating conditions for congestion and diffuse thrombosis of small DMVs. When the injury is severe, increased pressure may lead to rupture of the vein and substantial bleeding. In this study, there were five cases of GM-IVH in the group with abnormal DMVs, three of which were combined with CSVT. Perivenous hemorrhage was the cause of damage to DMVs in late preterm infants but was not the most important factor; congestion/thrombosis of DMVs may be isolated or associated with other lesions. Maternal preeclampsia, diabetes mellitus, chorioamnionitis, fetal distress, birth asphyxia, sepsis, heart defects and hereditary prethrombotic lesions are all high-risk factors for thrombosis (19–21). Central venous hypertension in the fetus, with circulatory heart failure, maybe the cause of DMV-related brain injury (22). We found that maternal gestational diabetes mellitus is a high risk factor for DMV lesions. Maternal diabetes in our study includes both gestational diabetes mellitus and antenatal diabetes mellitus. An immature coagulation system also increases the risk of thrombus in neonates (23), but in this retrospective analysis, we did not examine coagulation function.

Premature infants with severe brain injury often show obvious neurological symptoms after birth and we found that infants with abnormal DMVs were more likely to have convulsions. This group were also more likely to have more serious, grade IV, WMI.

The characteristic pattern of congestion and thrombosis in DMVs was largely reflected by neuroimaging, which showed linear white matter lesions with the same anatomical distribution as DMVs. Changes in low intensity signals in acute stage lesions indicated enlargement, hyperemia and/or thrombosis of DMVs, consistent with an earlier report (8). Lesions of the corpus callosum, the posterior limb of the internal capsule and the gray matter, such as thalamic injury, were more obvious in DWI in the early postnatal period. There were no significant characteristic signal changes in the T2-weighted images of infants with WMI, but normal DMVs, and the lesions were more apparent in DWI. WMI associated with DMV lesions was mainly concentrated in the prefrontal and frontal parietal areas, which is consistent with previous reports (8, 22, 24). The temporal area is less affected than other brain areas, probably because thrombi do not form easily in the thin white matter in the temporal area and the relatively short medullary vein. The transcerebral vein in the temporal lobe is also more developed, which is another reason why WMI occurs less easily. Some infants with periventricular confluent white matter injury did not show an extensive distribution, although there was a clear abnormal signal in the corpus callosum, which is an important part of the white matter (Figure 2). In this study, these cases, in which the corpus callosum was the main area of WMI, were included in the severe WMI group. In addition to the selective vulnerability of white matter in premature infants, the white matter may also have grade injury, which means that different degrees of the same type of damage may occur within a particular area. WMI in infants is usually not isolated; neurons in the thalamus are most often affected and the pontine is also commonly involved (25). In this study, infants with abnormal DMVs were more likely to have thalamic injury. When lesions occur in the DMVs, pressure in the medullary vein increases and, when there is insufficient decompression, disorders in thalamic vein drainage may lead to hemorrhagic or ischemic injury, which may differ from other mechanisms of WMI-induced thalamus involvement.

In infants with abnormal DMVs, a total of nine cases (60%) of PVL-like WMI were found on the MRI examination. Since the other six cases did not receive a second MRI scan, the true incidence of PVL-like changes may be higher than this. PVL-like changes also occurred in 10 infants (17.9%) with normal DMVs, indicating that severe WMI, especially WMI associated with congestion/thrombosis of DMVs, is an important cause of PVL-like WMI. PVL is, however, a pathological diagnosis and imaging cannot determine the original pathological mechanism and replace pathological diagnosis. Ultrasound has a very high sensitivity to detect cystic PVL and a good technique to screen and idenify PVL. MRI is more sensitive, depicts the non-cystic forms more accurately and can distinguish between the haemorrhagic and the non-haemorrhagic forms of PVL. Therefore, we can combine the advantages of ultrasound screening and MRI accuracy which helps to identify the cause of WMI and suggest new directions for treatment, management and prevention strategies.

### Limitation

One limitation of this study is that there were relatively few cases with abnormal DMVs, which makes it impossible to accurately determine the risk factors for development of this type of injury. Coagulation function was not measured and it is unclear whether congestion/thrombus of DMVs was associated with coagulation abnormalities. In this retrospective study, the lack of susceptibility weighted imaging (SWI) also means that some DMV lesions may have been missed, leading to research bias.

## Conclusion

In late preterm infants, hyperemia/thrombosis of DMVs is an important cause of WMI, especially PVL-like WMI. In future studies, we hope to include more infants, especially very premature infants, and to use SWI and other imaging techniques to build up a more appropriate imaging spectrum for PVL. It will also be helpful, in the future, to use MRI to determine whether or not WMI is associated with hemorrhagic injury, and to clarify differences in WMI distribution, gray matter injury and PVL outcome between hemorrhagic and non-hemorrhagic WMI.

## Data Availability Statement

The original contributions generated for this study are included in the article/supplementary materials, further inquiries can be directed to the corresponding author/s.

## Ethics Statement

The studies involving human participants were reviewed and approved by Shengjing Hospital of China Medical University Ethics Committee (Shenyang, China). Written informed consent to participate in this study was provided by the participants' legal guardian/next of kin.

## Author Contributions

DC and JM: study conception and design. DC, JS, QL, and WB: data acquisition. DC, JS, QL, WB, and JM: analysis and data interpretation. DC: drafting of the manuscript. JM: critical revision. All authors approved the final manuscript as submitted.

## Conflict of Interest

The authors declare that the research was conducted in the absence of any commercial or financial relationships that could be construed as a potential conflict of interest.
